# Atypical episodes of dissociation in an adolescent female - possible relationship with the menstrual cycle

**DOI:** 10.1192/j.eurpsy.2021.1668

**Published:** 2021-08-13

**Authors:** C.-E. Stoica, D. Cotuna, M. Pistea, A.-M. Ciubotariu, I. Mihailescu, F. Rad

**Affiliations:** Child And Adolescent Psychiatry, “Alexandru Obregia” Clinical Psychiatry Hospital, Bucharest, Romania

**Keywords:** dissociative disorder, acute onset, periodicity, psychosis

## Abstract

**Introduction:**

Psychiatric symptoms related with menstrual cycle vary from dysphoria to psychosis. There are only a few cases of menstrual psychosis reported, all characterized by acute onset, against a background of normality, brief duration, with full recovery and a circa-mensual periodicity.

**Objectives:**

We report a case of dissociative disorder, in a teenage girl, with atypical presentation and an unusual periodicity of symptoms and recoveries.

**Methods:**

Presentation of a case of dissociative disorder, followed by a review of the similar cases described in the literature.

**Results:**

We are presenting a case of a 15 years old female, who presented in our Emergency Department for confusion, anxiety, negativism in verbal and non-verbal response, bradylalia and bradypsychia, insomnia for over 48 hours. The symptoms suddenly began two days before arrival in our clinic. From the patient’s personal history, we retain the following: menarche at 14 years old, irregular periods, hypermenorrhea. Patient was born premature, G=1200g, spastic diplegia, periventricular leukomalacia (MRI – 2018). Three similar episodes happened a year ago, with one month periodicity, with spontaneous remission after 5-6 days. Patient was treated with antipsychotics and benzodiazepines for the second and the third episode, but the treatment was stopped six month ago. The investigations results were normal, except for a high level of plasmatic cortisol. Patient fully recovered in the day the menses stopped.

**Conclusions:**

We considered this case to be atypical due to the sudden debut and recovery and there are still some remaining questions. Is it hormonal related, menstrual related or is it exclusively a psychiatric condition?

**Disclosure:**

No significant relationships.

